# The Web-Based Pain-at-Work Toolkit With Telephone Support for Employees With Chronic or Persistent Pain: Protocol for a Cluster Randomized Feasibility Trial

**DOI:** 10.2196/51474

**Published:** 2023-10-30

**Authors:** Holly Blake, Wendy J Chaplin, Elaine Wainwright, Gordon Taylor, Paul McNamee, Daniel McWilliams, Victoria Abbott-Fleming, Jain Holmes, Aaron Fecowycz, David Andrew Walsh, Karen Walker-Bone

**Affiliations:** 1 School of Health Sciences University of Nottingham Nottingham United Kingdom; 2 Pain Centre Versus Arthritis University of Nottingham Nottingham United Kingdom; 3 NIHR Nottingham Biomedical Research Centre Nottingham United Kingdom; 4 Aberdeen Centre for Arthritis and Musculoskeletal Health (Epidemiology Group) School of Medicine, Medical Sciences and Nutrition University of Aberdeen Aberdeen United Kingdom; 5 College of Medicine and Health University of Exeter Exeter United Kingdom; 6 Health Economics Research Unit Institute of Applied Health Sciences University of Aberdeen Aberdeen United Kingdom; 7 Academic Unit of Injury, Recovery and Inflammation Sciences School of Medicine University of Nottingham Nottingham United Kingdom; 8 The Patient Voices Committee British Pain Society London United Kingdom; 9 School of Public Health and Preventive Medicine Monash University Australia United Kingdom

**Keywords:** eHealth, chronic pain, disability, workplace, randomized controlled trial, feasibility

## Abstract

**Background:**

Chronic or persistent pain affects one’s ability to work or be productive at work, generating high societal and economic burden. However, the provision of work-related advice and support for people with chronic pain is variable or lacking. The Pain-at-Work (PAW) Toolkit was cocreated with people who live with pain, health care professionals, and employers. It aims to increase knowledge about employee rights and how to access support for managing a painful chronic condition in the workplace and provides advice on lifestyle behaviors that facilitate the management of chronic pain.

**Objective:**

We aimed to establish the feasibility of conducting a definitive cluster randomized controlled trial comparing access to the PAW Toolkit and telephone support calls from an occupational therapist (PAW) with treatment as usual (ie, standard support from their employer). Our primary outcomes are establishing parameters of feasibility, acceptability, usability, and safety of this digital workplace health intervention. We will assess the candidate primary and secondary outcomes’ feasibility and test research processes for a definitive trial.

**Methods:**

This is an open-label, parallel 2-arm pragmatic feasibility cluster randomized controlled trial with exploratory health economics analysis and a nested qualitative interview study. We aim to recruit 120 participants from at least 8 workplace clusters (any type, >10 employees) in England. The recruitment of workplaces occurs via personal approach, and the recruitment of individual participants is web based. Eligible participants are vocationally active adults aged ≥18 years with internet access and self-reporting chronic pain interfering with their ability to undertake or enjoy productive work. A restricted 1:1 cluster-level randomization is used to allocate employment settings to PAW or treatment as usual; participants are unblinded to group allocation. Following site- and individual-level consent, participants complete a web-based baseline survey (time 0), including measures of work capacity, health and well-being, and health care resource use. Follow-up is performed at 3 months (time 1) and 6 months (time 2). Feasibility outcomes relate to recruitment; intervention fidelity (eg, delivery, reach, uptake, and engagement); retention; and follow-up. Qualitative evaluation (time 2) is mapped to the Capability, Opportunity, Motivation–Behavior model and will explore intervention acceptability to employees and employers, along with individual and contextual factors influencing the delivery and uptake of the intervention.

**Results:**

Ethics approval was obtained in March 2023. Trial recruitment began in June 2023.

**Conclusions:**

The PAW Toolkit is the first evidence-based digital health intervention aimed at supporting the self-management of chronic or persistent pain at work. This study will inform the design of a definitive trial, including sample size estimation, approaches to cluster site identification, primary and secondary outcomes’ selection, and the final health economic model. Findings will inform approaches for the future delivery of this digital health intervention.

**Trial Registration:**

ClinicalTrials.gov NCT05838677; https://clinicaltrials.gov/study/NCT05838677

**International Registered Report Identifier (IRRID):**

DERR1-10.2196/51474

## Introduction

### Background

In the United Kingdom, chronic or persistent pain affects approximately one-third to one-half of the population [1,2]. This figure is predicted to increase largely owing to an aging population [1-3]. Chronic pain poses a high societal and economic burden [4,5]. In England, the direct medical costs of chronic pain amount to approximately £580 million (US $707.6 million), including the total prescription of analgesic medication and pain-related primary care appointments [6]. The broader economic costs to individuals with chronic pain, their employers, and society are substantial because of the costs of health and social care, productivity losses, sickness absenteeism, and early retirement [7,8], estimated to be over £100 billion (US $122 billion) annually [7]. People living with chronic pain report significant impacts on physical and mental health [9] and lower quality of life than the general population and patients with other long-term conditions [10]. The COVID-19 pandemic has increased the overall burden of chronic pain worldwide through the emergence of newly diagnosed conditions and by exacerbating existing conditions or their risk factors [11-13].

The importance of promoting and improving the way in which people self-manage chronic pain conditions is advocated in clinical guidelines [14]. Self-management interventions can be effective in improving pain, mental health, and health-related quality of life outcomes [15]. However, self-management interventions for chronic pain often focus on specific conditions or pain types (eg, *back pain* [16-20], *chronic musculoskeletal conditions* [21,22], *arthritis* [18,23], *chronic orofacial pain* [24], and *cancer pain* [25]). This potentially excludes a broader spectrum of chronic primary or secondary pain conditions (as in Korwisi et al [14]) and individuals who experience pain but lack a medical diagnosis or do not access health care services.

Studies on the management of chronic pain commonly focus only on medical, physical, or psychological strategies for pain management, whereas very few studies have reported on work-related impacts, strategies, or outcomes [26]. However, interventions focused on changes at work for people with chronic conditions (eg, to working conditions, work environment, and work organization) may enhance work participation across a range of chronic diseases [27]. Chronic pain affects people’s ability to be productive at work, be fulfilled at work, or remain in the active workforce [9,28-30] and leads to social inequalities (eg, *disability pay gap*). Retaining vocationally active adults in the workforce is important for reducing health and social inequalities because employment is inversely related to pain severity [31] and worklessness is associated with poorer physical and mental health [8,32,33], social exclusion [34], and all-cause mortality [35].

In clinical services, access to work-related advice (eg, occupational therapy) for people with chronic pain is highly variable and influenced by many factors such as referrals, provider availability, and resources [36]. Occupational therapists (OTs) work within health care and occupational health settings, providing interventions for pain management and return to or staying at work, but access to OTs is limited [37].

Researcher-led interventions targeting work-related outcomes are promising (eg, *vocational rehabilitation* [38,39]), but such studies tend to focus on specific conditions (eg, arthritis) and recruit participants from clinical settings (eg, rheumatology clinics), excluding people with other chronic pain conditions and those who are self-managing their condition outside of health care services.

Similar to self-management interventions, workplace-delivered interventions for the management of chronic pain also tend to focus on specific conditions (eg, *back pain* [40-45], *neck pain* [46-48], *shoulder pain* [49,50], and *musculoskeletal conditions* [51-54]); target specific occupational groups or job types (eg, *workers with physically demanding work* [52] or *nurses* [43]); or focus on specific types of intervention (eg, *exercise or physical activity* [41,42,51,55], *physical conditioning* [44], *rehabilitation interventions* [45,56], *ergonomic interventions* [57,58], and *return-to-work or retention interventions* [59-64]). Therefore, many workplace-delivered interventions will not reach the wider population of adults with chronic pain (with or without a formal diagnosis) or the wider spectrum of occupational groups. In addition, the narrow focus of existing interventions means that people with chronic pain need to access information and support from multiple places and may not know where and how to access it.

In practice, employers do not routinely provide support for and advice to employees with chronic pain conditions [65], and the provision of education and supportive materials for employees with chronic pain is therefore inconsistent or lacking across organizations and sectors. Only 30% to 34% of the UK workforce has access to specialized occupational health care [66]; a more recent estimate of 51% with access [67] was argued to be vastly overestimated [68]. Even when occupational health services are available, some occupational health professionals are not necessarily knowledgeable about chronic pain [69].

Digital approaches to the delivery of self-management are gaining popularity. Immersive technologies such as mobile health (the use of mobile phones and other wireless technology) and eHealth (the use of information and communication technology to support health and health care) have been used to provide pain therapy, education, symptom monitoring, lifestyle advice, health coaching, and cognitive behavioral therapy via virtual reality, mobile apps, or web-delivered programs in adults with diverse pain conditions [70-86]. Such interventions are primarily focused on improving pain, functional disability, or psychological outcomes but do not address barriers to work, facilitators of work ability, or pain self-management in the context of work, and work-related outcomes are often not measured.

In summary, there is a clear need for workplace interventions aimed at building the knowledge, skills, and confidence of vocationally active adults to effectively self-manage their condition at work (eg, through help seeking, adjusting job roles or physical environments, accessing support, and healthy lifestyle behaviors). Intervention is required that delivers comprehensive advice and support across a range of self-management areas, which is suitable for employees with any type of chronic pain working in any type of employment setting. Digital solutions are a potentially low-cost and scalable approach for the delivery of health interventions [87]. They have wide geographic reach and offer flexibility to the end user, which is increasingly valuable in the context of changes in job roles, work patterns, and locations (eg, hybrid or remote working) that have escalated in recent years [88,89].

The Pain-at-Work (PAW) Toolkit is the first accessible digital resource [65,90] designed to support people with chronic or persistent pain in self-managing their condition at work. It is designed to be relevant to any vocationally active adult with chronic pain in any organization type, size, or sector. The PAW Toolkit offers evidence-based advice about chronic or persistent pain, disability rights, work capacity, pain self-management strategies, and signposting to support. The design of the PAW Toolkit considers known enablers and barriers to engagement in digital interventions for people with chronic pain (eg, flexibility for access, inclusivity for people with disabilities, and low technological skill requirement). This intervention has been cocreated, pilot-tested, and evaluated with employees from public, private, and third sector organizations across the United Kingdom, and the comprehensive development processes are described elsewhere [65]. The feasibility and acceptability of the PAW Toolkit across employment sectors and different organization sizes and types, and the feasibility of testing the PAW Toolkit within a trial is yet to be determined.

This study is an important next step toward establishing the effectiveness and cost-effectiveness of the PAW Toolkit as a workplace intervention to support employees with chronic pain. Ultimately, the PAW Toolkit could contribute to reducing social inequalities (ie, *disability pay gap*) and the overall health, societal, and economic burden of chronic pain.

### Aims and Objectives

The overall aim of the study is to determine the feasibility of conducting a definitive cluster randomized controlled trial (cRCT) on the effectiveness and cost-effectiveness of the PAW Toolkit with telephone support for vocationally active adults with chronic or persistent pain.

To achieve this, the objectives are as follows:

1. To measure feasibility outcomes to assess whether it would be possible to recruit to a definitive trial (recruitment and retention)
2. To test the feasibility of reaching different employee groups (eg, age, gender, ethnicity, and job role or type), sectors (eg, public, private, and third), and organizational types (eg, small to medium or large enterprises)
3. To explore whether participants and employers find the intervention and trial design acceptable
4. To obtain an estimate of the intracluster correlation coefficient to inform the future sample size calculation for the main trial
5. To collect a range of outcome measures to help identify the most appropriate primary outcome for a definitive trial
6. To assess the feasibility of capturing health economic data in a future trial
7. To design a future trial and implementation plan

## Methods

### Trial Design

This study is an open-label, 2-arm multicenter pragmatic cluster randomized controlled feasibility trial of the PAW Toolkit compared with a no-intervention control group in working adults with chronic or persistent pain. Both groups will continue to receive treatment as usual (TAU; ie, standard support from their employer). The analysis will be performed on an intention-to-treat basis. The feasibility trial included an exploratory health economics evaluation and a nested qualitative interview study. The study aligns with the Medical Research Council framework for developing and testing complex interventions [91] and will be conducted in accordance with the CONSORT (Consolidated Standards of Reporting Trials) extension to randomized pilot and feasibility trials [92]. The protocol was developed using the SPIRIT (Standard Protocol Items: Recommendations for Interventional Trials) guidelines [93].

### Ethical Considerations

The University of Nottingham Faculty of Medicine and Health Sciences Research Ethics Committee granted ethics approval on March 31, 2023 (Faculty of Medicine and Health Sciences 237-0323). The trial was prospectively registered on May 1, 2023 (ClinicalTrials.gov NCT05838677).

### Patient and Public Involvement

Our extensive preparatory work to inform the PAW Toolkit development (n=472) was a rigorous collaborative participatory process involving surveys with employees (n=274) and employers (n=107), a stakeholder workshop (n=27), and expert peer review (n=40) [65]. Content was cocreated with “Burning Nights” (a pain charity in the United Kingdom), people living with chronic pain, health care professionals, occupational health and trade union advisers, and employers. Our trial protocol was discussed with 6 members of the patient and public involvement groups at 2 national pain centers. People with lived experience of chronic pain are represented in our trial management group, trial steering group, and trial advisory group. This paper is coauthored by the chair of The Patient Voices Committee of the British Pain Society.

### Eligibility Criteria

#### Settings

Organizations are eligible if they are located in England, are from any sector (public, private, or third), and have ≥10 employees (small: 10-49 workers; medium: 50-249 workers; or large: >250 workers). At least 8 organizations will be recruited as the unit of randomization.

#### Participants

Participants are eligible if they are working-age adults (employees), are aged 18 years and older, have self-reported chronic pain interfering with their ability to undertake or enjoy productive work, can comprehend English language, and are able to provide informed consent. We will include employees of any age, gender, nationality, ethnicity, income level, occupation (eg, manual or office based, low or high skilled, and low or high income), or employment status (eg, full or part time, permanent, contracted or subcontracted, volunteer, and gig workers). We use the term “employee” in this protocol to cover any type of worker. Organizations are excluded if they are outside England or are micro-organizations with fewer than 10 employees. Participants are excluded if they do not identify as having chronic pain, are unemployed at the time of recruitment, or are unable to provide informed consent. Employees with an inability to comprehend written English are excluded because it is a requirement to provide informed consent and understand the current PAW Toolkit materials. Although access to the internet is a prerequisite to engage in the study (to be able to use the intervention and complete data collection surveys), we will record the number of participants requiring telephone support for survey completion or challenges with intervention use as a proxy indicator of computer literacy.

### Sample Size

A formal sample size calculation is not required for feasibility studies, although based on prior studies with a similar design [94-96], our aim is to recruit 120 employees from at least 8 organizations. Given the variability in organization size, if 120 employees are not recruited from 8 organizations, additional organizations may be recruited. Owing to the variability in the number of employees agreeing to participate in individual organizations, the total number of employees may exceed 120.

### Study Procedure

#### Overview

**Figure 1:**
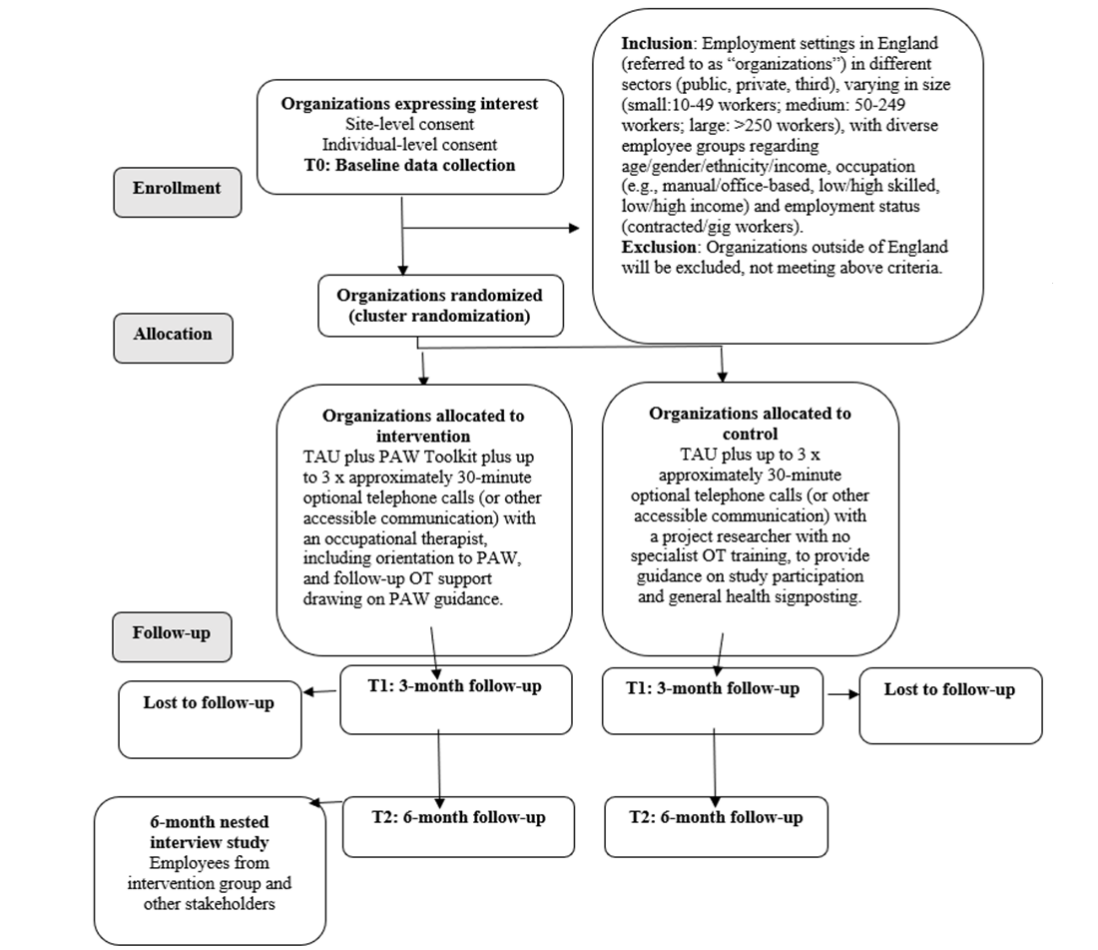
CONSORT (Consolidated Standards of Reporting Trials) participant flow diagram. TAU refers to services and provisions provided as routine practice by the participating organization. OT: occupational therapy; PAW: Pain-at-Work; TAU: treatment as usual; T0: time 0 (baseline); T1: time 1 (3 months); T2: time 2 (6 months).

The recruitment of organizations (cluster sites) and employees (participants) will be undertaken by the study researcher on a rolling basis. Each participant will be involved for approximately a 6-month duration for the feasibility trial, comprising a 6-month intervention period (baseline to final follow-up). In a subsample, qualitative interviews will be conducted at 6 months and within 8 weeks of the study end. The participant’s journey through the study is shown in the CONSORT flow diagram in Figure 1. Participants will remain free to withdraw from the trial at any time without giving reasons and without prejudicing their employment or health care and will be provided with a contact point where they may obtain further information about the trial.

#### Recruitment of Organizations

Organizations in England will be identified in several ways by promoting information about the study via employer professional networks and platforms (eg, Building People), learned societies and professional organizations (eg, as identified on GOV.UK [97]), and social media (eg, LinkedIn and X [formerly known as Twitter]). Eligible organizations will be sent a formal invitation to participate by email containing a weblink to a study information sheet and consent form, and reminders will be sent to nonresponders. They will have the opportunity to discuss the study (eg, by telephone or videoconferencing) with a member of the project team. Once organizations volunteer to participate, the researcher will review the organization against the eligibility criteria to confirm that the organization meets the entry criteria. Web-based informed consent is obtained from the employer representative (“gatekeeper”). Information about the organization (eg, type, size, sector, and number of employers) will be collected by the researcher via email, telephone, or videoconference call. Gatekeepers will then be asked to provide information about the study to their employees by whichever method is appropriate for their organization. Employers’ routes to providing study information to their employees will be recorded. As this is a feasibility study, strategies to incentivize organizations to participate may be added if uptake is low.

#### Recruitment of Participants

Employees receive information about the study from their employers (eg, email or other employer-selected routes). Employees will self-determine whether they meet the eligibility criteria as the presence of chronic or persistent pain conditions may only be known to individuals. Those who wish to participate in the study will independently access the study information via a weblink that they will receive via email, provide their consent, and complete baseline measures via a web-based data collection form. Employees will receive an email confirmation of their participation and be assigned a unique identifier. Participants will be able to contact the project researcher (by email, telephone, or videoconferencing) with any queries related to their participation in the trial or to access support with completion of the web-based surveys.

### Stopping Guidelines

Employees will be made aware that they can withdraw their consent at any time during the trial without it affecting their employment. If a participant chooses to leave the study prematurely, the primary reason for discontinuation will be determined and recorded if possible. Participants who have withdrawn will not be replaced. They will be made aware (via the information sheet and consent form) that should they withdraw, the data collected to date cannot be erased and may still be used in the final analysis. In the unlikely event that an organization withdraws participation after randomization, the organization will not be replaced. The organization will be made aware that the data collected on the organization and employees at that organization to date cannot be erased and may still be used in the final analysis. Participants will be advised that their organization has withdrawn.

### Randomization

Organizations are randomized by the study statistician to either (1) active control group or (2) the PAW Toolkit with telephone support. Cluster randomization (rather than individual randomization) is required because of the nature of the intervention and risk of contamination between intervention and control participants working in the same organization. Organizations are randomized into the intervention or control group using an allocation ratio of 1:1. As this is an eHealth intervention, the researcher and participants will not be blinded to group allocation.

### Interventions

#### Active Control Group

Participants will not receive the PAW Toolkit but continue to receive TAU from their employer. The nature of TAU will be recorded as part of the feasibility study. Depending on the employing organization, TAU may consist of but is not limited to any combination of the following: occupational health, counseling, line manager support, and signposting to education about factors that may have positive or negative effects on chronic pain. To help minimize the possibility of outcomes being influenced simply by the additional contact time offered to participants in the intervention group (ie, social support provided by additional telephone calls or contacts), this is an active control group. In this study, active control means that participants can access up to 3 opt-in nonspecialist telephone calls from a researcher for general discussion (eg, their participation in the study), by means of providing a comparable level of social contact that is unrelated to the intervention.

#### PAW Toolkit

Intervention participants will receive TAU and the web-based PAW Toolkit. The PAW Toolkit is designed to be relevant to any employee with chronic pain in any organization type, size, or sector. PAW offers evidence-based advice on chronic or persistent pain, disability rights, work capacity, pain self-management strategies, and signposting to support. It is based on a theory of change: “Providing employees with access to the PAW Toolkit will increase knowledge about employee rights, how to access support for managing a painful chronic condition in the workplace, and lifestyle behaviours that facilitate the management of chronic or persistent pain. This in turn will lead to improved self-management of pain at work. The ultimate aim is to improve outcomes for individuals (self-efficacy, work ability, job perceptions, health, and wellbeing) and organisations (presenteeism, absenteeism)” [65].

The PAW Toolkit is authored by the lead author (HB) and colleagues (Sarah Greaves, Sarah Somerset, and VA-F) [90]. External peer review was undertaken from January to February 2023 to ensure that the materials were current, and minor updates were completed in April 2023. It is free to access, and participants will not be paid to access it during the trial. The front page includes logos for the institutions that developed the intervention and funded its development, including a university, a pain charity, and a research council. The toolkit is based on direct instruction (information and advice) and experiential learning (advice being acted on by the end user). No training is required to use the PAW Toolkit. Individuals involved in the development processes (eg, stakeholder consultation, peer review, and technical support, as reported in the study by Blake et al [65]) are named in the PAW Toolkit. The pages contain brief text, images, multimedia (ie, video clips), and hyperlinks.

**Figure 2:**
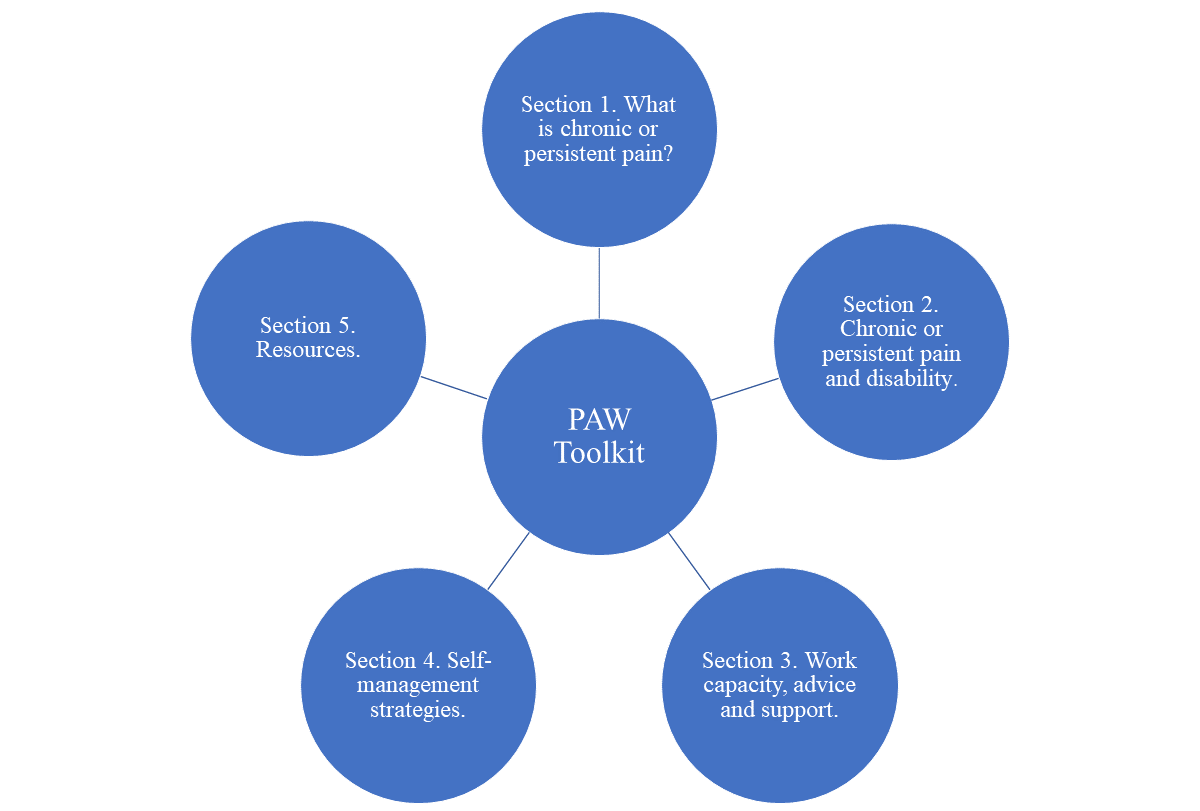
Pain-at-Work (PAW) Toolkit sections.

The PAW Toolkit is accessed via a weblink [90]. Each organization participating in the trial will receive a unique link from the study participants recruited from their site. Technical support is available throughout the trial to resolve any arising technical issues. The delivery of the toolkit is asynchronous (ie, not prescheduled and flexible access at a time to suit the end user). Although the PAW Toolkit may be used as a stand-alone intervention, in this feasibility trial, the delivery is supported by up to 3 opt-in OT telephone appointments (approximately 30 min of contact time each) or other accessible communication if requested by a participant, such as SMS text messaging. OT support involves orientation to the PAW Toolkit, individually tailored advice, signposting, and behavioral strategies for managing pain at work, which are aligned with the PAW Toolkit content. Content headings are shown in Figure 2. The sections and contents of the PAW Toolkit are described in Multimedia Appendix 1 [65]. The TIDieR (Template for Intervention Description and Replication) checklist and guide [98] is used to describe the intervention and use parameters (ie, dose, frequency, and duration) in Multimedia Appendix 2.

**Figure 3:**
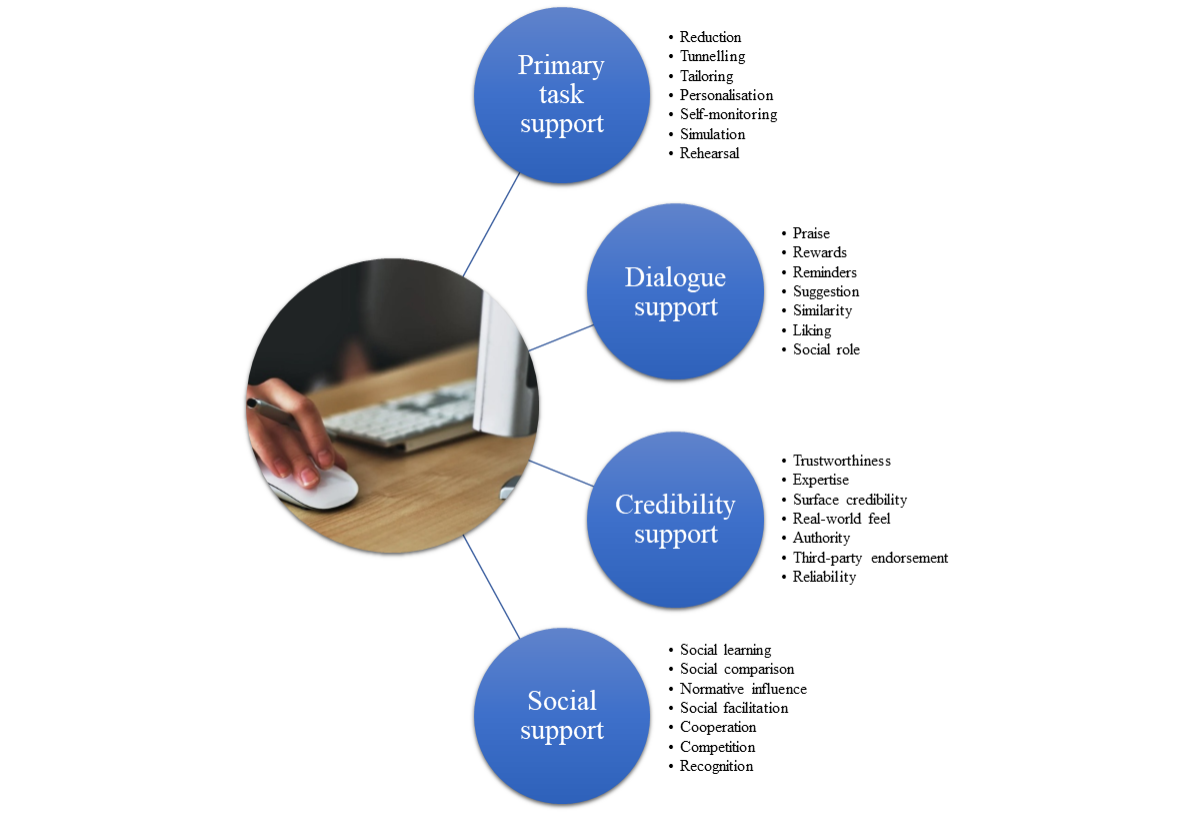
Persuasive system design.

The intervention draws on the principles of persuasive system design [99] (Figure 3). Detailed mapping of the PAW Toolkit intervention and feasibility trial to the persuasive systems design is described in Multimedia Appendix 3.

Evaluation of the intervention will draw on the Technology Acceptance Model [100] and behavior change theory (Behavior Change Wheel and Capability, Opportunity, Motivation–Behavior [COM-B] model [101]).

### SMS Text Message Reminders

Participants in both the control and intervention groups will receive SMS text message reminders delivered through an automated system because SMS text messages can improve responses to web-based data collection surveys [102]. For intervention participants only, messages will include reminders to access the PAW Toolkit and OT support, as SMS text message reminders can increase the uptake of health interventions [103]. Message content will be informed by the COM-B model [101], an approach used previously for SMS text messaging aligned with workplace health intervention [104]. The content, frequency, and duration of messages will be determined through patient and public involvement consultation, message peer review, and prior research.

### Study Outcomes

Outcomes are separated into (1) those that determine the feasibility and acceptability of a large definitive trial, (2) employer outcomes, and (3) participant-reported outcome measures (PROMs) to inform the choice of the primary outcome for the definitive trial. Outcomes will be measured at baseline (time 0: T0), 3 months (time 1: T1), and 6 months (time 2: T2). Items from the Checklist for Reporting Results of Internet E-Surveys (CHERRIES) [105] will be applied when reporting the findings from our web-based surveys. Multimedia Appendices 4 and 5 provide details of all feasibility and acceptability (Multimedia Appendix 4), employer- and participant-reported (Multimedia Appendix 5) data collection measures, and timeframes.

### Employer-Reported Data

At the time of recruitment, details about the employment setting (Multimedia Appendix 5) will be collected from the organization representative (“gatekeeper”). This will document the sector, organization type, and size; number of staff; role of the “gatekeeper” employee representative; views toward workplace culture at the organization; and description of TAU in terms of existing provisions to support staff with long-term health conditions. Sickness absence data will be requested from the organization’s records, with consent from the participants at T0, T1, and T2.

### PROMs Data

#### Overview

PROMs (Multimedia Appendix 5) are self-assessed using web-based questionnaires, which include logos for the lead institution (university) and trial funder (charity). Closed, web-based measures will be collected using the Jisc web-based surveys at T0, T1, and T2. To help minimize attrition, participants completing surveys at all 3 time points from both groups will have the opportunity to opt into a prize draw to receive a £250 (US $305) high street shopping voucher. Participants will provide sociodemographic data (ie, age, gender, ethnicity, income, and education); health data (ie, pain conditions, present numeric pain rating scale [0-10], comorbidities, and medications); employment characteristics (eg, employment status, occupation, hours worked, and job features); sector; size and type of employing organization; assessment of their perception of organization culture; and the TAU services or support they have accessed via their employer. TAU may consist of but is not limited to any combination of the following: occupational health, counseling, line manager support, and signposting to education about factors that may have positive or negative effects on chronic pain.

PROMs are collected via a web-based survey at T0, T1, and T2 to measure the changes between time points, as presented in the following sections.

#### Work-Related PROMs

The Work Limitations Questionnaire-25 [106] is used as a measure of work presenteeism, that is, the “degree to which health problems interfere with specific aspects of job performance and the productivity impact of these work limitations” [107]. The scale demonstrates high reliability and validity in employee populations with chronic conditions [106-108].

The Work Productivity and Activity Impairment Questionnaire: General Health V2.0 [109] is used to measure absenteeism, presenteeism, work productivity loss, and activity impairment during the past 7 days. The validity, reliability, and responsiveness of the scale have been demonstrated in adults with chronic conditions [110-112].

The Work Ability Index Item 1 [113] is used to measure work ability (ie, how well an employee is able to perform their work). Validity and reliability have been demonstrated in employee populations [114].

The Work Self-Efficacy Scale [115] is used to measure employees’ perceptions of their capability to manage specific work domains. The scale demonstrates good psychometric properties [116].

Single global items will be used to measure job satisfaction [117] and job stressfulness [118]. These items have established reliability and validity [117,118] and have been used in other employee populations [119]. Turnover intentions will be assessed using a single global item used in a web-based survey with an employee population adapted from [117] and used in [119].

Social support in the workplace will be measured using the Demand Control Support Questionnaire Social Support Subscale [120]. The scale has been shown to be valid and reliable in workplace samples [120].

#### Psychological and Health-Related Quality of Life PROMs

Depression symptoms will be measured using the Patient Health Questionnaire [121]. The scale has established validity as a screening tool for major depression [122,123].

Anxiety will be assessed using the General Anxiety Disorder Scale [124,125]. The scale has established reliability and validity [124,126,127], including in employee samples [123,128].

Health-related quality of life will be measured using EQ-5D-5L [129]. The scale has been shown to be reliable and valid for use in people with chronic conditions [130,131].

#### Health Resource Use

Exploratory health economics data capture will be informed by previously published guidance produced by a coauthor (PM) on designing and undertaking a health economics study of digital health interventions [132]. A health care resource use questionnaire will be tested to measure completion rates for items of resource use, such as frequency of use of secondary and primary care, social care, private health care, and medications. Items are adapted from health economics data capture in previous research with pain populations [133,134].

#### Technology Adoption PROMs

At T1, the intervention group will complete the questions related to technology adoption. Items were developed by the study team and mapped to the Technology Acceptance Model [100] to measure perceived knowledge, perceived ease of use, perceived understandability, attitudes, and behavior related to the intervention. This will provide data to assess whether and how participants engaged with this digital health intervention and provide insights into the likely impacts of this engagement.

### Data Analysis

The analysis will primarily be descriptive in line with the aims of a feasibility study. This will include estimates of recruitment and retention rates and descriptive statistics for baseline and follow-up outcome measures. For PROMs, we will undertake a dummy-run analysis presenting total scores (T0 and T1) and change from T0 to T2 (adjusting for baseline). Differences between arms may be presented with 95% CIs, where appropriate. Health economic measures (TAU costs, health care resource use costs comprising medicine use, primary care consultations, hospital stays, and outpatient visits for reasons related to chronic pain, anxiety, or depression) and quality of life measurement using EQ-5D-5L will be presented as unadjusted mean (SD) and median (IQR) values. Regression-based adjusted costs and EQ-5D-5L utility values will also be calculated, with baseline cost, quality of life values, treatment group, PAW Toolkit engagement (time spent), work absence, and respondent demographics as covariates. The percentage of missing data per resource use item and EQ-5D-5L dimension will also be presented. This analysis, along with a framework analysis of patients’ views on the relevance of PROMs to self-management of their chronic pain condition (captured as a part of the questionnaires and nested interviews), will guide the choice of relevant PROMs for use in a definitive trial of the PAW Toolkit.

### Nested Qualitative Interview Study

At T2, we will conduct semistructured individual interviews with up to 40 stakeholders from intervention sites to explore the views of employees who received the intervention and key stakeholders that employees identify as involved in their support (eg, line managers, company owners, human resources, occupational health, or trade union). Participants will be purposively selected to reflect diverse views from across self-identified employee groups (eg, age, gender, ethnicity, and job type), sectors, organization size, and type. The eligibility criteria for the nested interview study are (1) working for an organization participating in the trial and selected for participation in the nested interview study (employees) and (2) employed in a role providing management or support for employees in a participating organization (stakeholders). The interviews will be held by telephone or videoconferencing (eg, Teams [Microsoft Corporation]) at a mutually convenient time and will be audio recorded with consent. The recordings will be transcribed in full and anonymized. We expect the interviews to last approximately 45 to 60 minutes.

Questions will be developed using a framework for qualitative research in feasibility randomized controlled trials [135,136] and reviewed by people with lived experiences of pain. Interviews will ascertain participants’ views about the feasibility and acceptability of the intervention, trial processes and outcome measures, and any perceived changes in individual or organizational outcomes. Employee questions are mapped to the COM-B model [101] to explore influencers of capability, opportunity, and motivation to self-manage their condition at work (including knowledge, attitudes, and confidence).

Employees and other stakeholders will provide informed consent via a web-based consent form. Verbal consent will be audio recorded before the interview. The qualitative researchers undertaking the interviews will provide information to the participants, explain the study, and obtain consent. It will be explained to the potential participant that entry into the study is entirely voluntary and that their employment will not be affected by their decision. Using framework analysis [137], we will explore barriers to or facilitators of engagement with or use of the intervention and recommendations for future implementation.

## Results

We received funding from the Nuffield Foundation and Versus Arthritis, and the project started in March 2023. The trial was opened for recruitment in June 2023. The goal is to recruit approximately 8 organizations and 120 eligible participants (approximately 60 in each arm). As of August 11, 2023, three organizations have been recruited and randomized. The trial is currently in the recruitment phase. Data collection is expected to be completed by August 2024. Data analysis will begin once all the data have been obtained, following the established plan.

## Discussion

### Overview

The aim of this study is to determine the feasibility of conducting a definitive cRCT on the effectiveness and cost-effectiveness of the PAW Toolkit with telephone support for vocationally active adults with chronic or persistent pain. The ultimate aim is to retain vocationally active adults in the workforce, which is important for reducing health and social inequalities. This feasibility trial will ascertain whether it is possible to recruit and retain organizations and eligible participants in a cluster randomized trial and provide insights into the feasibility of reaching different types of organization (of diverse sizes, types, and sectors) and employees (of diverse occupational and demographic groups). The data will determine whether the intervention and trial processes are acceptable to employers and employees. The knowledge gained from the study will inform the design of a definitive trial, including sample size estimation, approaches to cluster site identification, selection of primary and secondary outcomes, and the final health economic model. This trial will ascertain how the PAW Toolkit can be best optimized in future research and implementation in real-world employment settings.

Our study addresses the limitations of prior studies on (1) self-management of chronic conditions, (2) workplace-delivered interventions for the management of chronic pain, and (3) studies that target work-related outcomes. Through this trial, we provide an intervention that delivers comprehensive advice and support across a range of self-management areas, which is suitable for employees with any type of chronic pain working in any type of employment setting.

### Limitations

We are unable to collect data from employees who do not participate in the trial. Workplace research presents challenges in terms of recruitment and high risk of attrition [138]. However, we have also included strategies intended to maximize uptake and retention.

### Conclusions

The PAW Toolkit is the first evidence-based digital health intervention aimed at supporting the self-management of chronic or persistent pain at work in vocationally active adults. The PAW feasibility trial will provide novel evidence on the feasibility of a cRCT evaluation of this digital intervention to support vocationally active adults at work, who are living with chronic or persistent pain.

## Appendix

Multimedia Appendix 1 Pain-at-Work Toolkit sections and content.

Multimedia Appendix 2 Application of the TIDieR (Template for Intervention Description and Replication) checklist to the intervention.

Multimedia Appendix 3 Mapping the Pain-at-Work Toolkit intervention and feasibility trial to persuasive systems design.

Multimedia Appendix 4 Feasibility and acceptability measures.

Multimedia Appendix 5 Employer- and participant-reported outcomes.

Multimedia Appendix 6 Peer-review report by the Nuffield Foundation’s Oliver Bird Fund and Versus Arthritis.

## Abbreviations

CHERRIES: Checklist for Reporting Results of Internet E-Surveys
COM-B: Capability, Opportunity, Motivation–Behavior
CONSORT: Consolidated Standards of Reporting Trials
cRCT: cluster randomized controlled trial
OT: occupational therapist
PAW: Pain-at-Work
PROM: participant-reported outcome measure
SPIRIT: Standard Protocol Items: Recommendations for Interventional Trials
TAU: treatment as usual
TIDieR: Template for Intervention Description and Replication
