# Supplementary material for: The Web-Based Pain-at-Work Toolkit With Telephone Support for Employees With Chronic or Persistent Pain: Protocol for a Cluster Randomized Feasibility Trial
Source: JMIR Res Protoc. 2023 Oct 30;12:e51474. doi: 10.2196/51474 (PMC10644198; doi:10.2196/51474)
Supplement: Multimedia Appendix 1 [file resprot_v12i1e51474_app1.docx]

**Multimedia Appendix 1.** PAW Toolkit sections and content (reproduced from from Blake et al, 2022 [65], which is published under Creative Commons Attribution 4.0 International License^a^).

| **Section 1. What is chronic or persistent pain?**  This section includes information to help employees understand what chronic or persistent pain is, how commonly people are affected in the UK, and how it significantly impacts people in different ways. A podcast is included with a personal story about someone’s experience of managing chronic pain in the workplace. |
| --- |
| **Section 2. Chronic or persistent pain and disability.**  This section includes information about the UK Equality Act 2010 and the legal definition of disability, to help employees to know and understand their statutory rights in the workplace. It also covers discrimination and harassment in the workplace and how to get help from organisations offering impartial support to employees. |
| **Section 3. Work capacity, advice and support.**  This section provides information and advice to employees about how to get the support they need at work to be able to do their job. It includes how to speak with an employer and understanding and accessing reasonable adjustments in the workplace. Examples of reasonable adjustments from people’s personal experiences are shared. The challenges of returning to work after a long-term absence are highlighted and the support available through phased returns and workplace adaptations are explained, including the different roles of occupational health services and the UK Access to Work services in accessing and supporting employees at work. |
| **Section 4. Self-management strategies.**  This section includes a variety of supported self-management resources to help with managing chronic or persistent pain. The information and resources provided by different expert organisations are aimed at supporting physical and mental health. The section provides an interactive guide to living well with pain. Information and resources provided support the understanding and management of stress, emotions, and practical skills, including pacing, goal setting, keeping physically active, relaxation and mindfulness skills. Short videos highlight people’s lived experiences of accessing different support to manage their chronic or persistent pain, including psychological support and information on how to access psychological therapies. |
| **Section 5. Resources.**  In addition to resources provided in sections 1-4, Section 5 provides access to further websites and apps to support a range of self-management advice and tools. |

^a^ Attribution 4.0 International (CC BY 4.0). Creative Commons. URL: https://creativecommons.org/licenses/by/4.0/ [accessed 2018-10-03]
