# Supplementary material for: The Web-Based Pain-at-Work Toolkit With Telephone Support for Employees With Chronic or Persistent Pain: Protocol for a Cluster Randomized Feasibility Trial
Source: JMIR Res Protoc. 2023 Oct 30;12:e51474. doi: 10.2196/51474 (PMC10644198; doi:10.2196/51474)
Supplement: Multimedia Appendix 2 [file resprot_v12i1e51474_app2.docx]

**Multimedia Appendix 2.** Application of TIDieR checklist to the intervention.

| **BRIEF NAME** |  |
| --- | --- |
| Provide the name or a phrase that describes the intervention. | The Pain-at-Work (PAW) Toolkit |
| **WHY** |  |
| Describe any rationale, theory, or goal of the elements essential to the intervention. | Theory of change: Providing employees with access to the PAW Toolkit will increase knowledge about employee rights, how to access support for managing a painful chronic condition in the workplace, and lifestyle behaviours that facilitate the management of chronic or persistent pain. This in turn will lead to improved self-management of pain at work.  The ultimate aim is to improve outcomes for individuals (self-efficacy, work ability, job perceptions, health and wellbeing) and organisations (presenteeism, absenteeism) |
| **WHAT** |  |
| Materials: Describe any physical or informational materials to be used in the intervention, including those provided to participants or used in intervention delivery or in training of intervention providers. Provide information on where the materials can be accessed (e.g., online appendix, URL). | The PAW toolkit is a digital package that was created in Xerte, an open-source software for authoring learning objects. It provides (a) evidence-based guidelines and signposting around work-capacity advice and support; (b) self-management strategies around working with chronic or persistent pain, (c) promotion of healthy lifestyles, and quality of life at work; (d) advice on adjustments to working environments and workplace solutions to facilitate work participation. Content and presentation were developed to consider known enablers and barriers to engagement in digital interventions for people with chronic pain, through flexibility for access, inclusivity for people with disabilities, and low technological skill requirement.  The toolkit provides evidence-based guidelines and signposting in five broad areas:   1. What is chronic or persistent pain? 2. Chronic or persistent pain and disability. 3. Work capacity, advice, and support. 4. Self-management strategies. 5. Resources.   It can be accessed at: <https://www.nottingham.ac.uk/toolkits/play_24452> (temporarily removed from public view while it is being tested in a feasibility trial). |
| Procedures: Describe each of the procedures, activities, and/or processes used in the intervention, including any enabling or support activities. | The ‘pre-planned schedule’ consists of five sections which is considered the full ‘dose’ of intervention content, designed to be accessed in succession from [Section 1](https://www.mdpi.com/2227-9032/10/1/56#sec1-healthcare-10-00056), [Section 2](https://www.mdpi.com/2227-9032/10/1/56#sec2-healthcare-10-00056), [Section 3](https://www.mdpi.com/2227-9032/10/1/56#sec3-healthcare-10-00056), [Section 4](https://www.mdpi.com/2227-9032/10/1/56#sec4-healthcare-10-00056) and [Section 5](https://www.mdpi.com/2227-9032/10/1/56#sec5-healthcare-10-00056). |
| **WHO PROVIDED** |  |
| For each category of intervention provider (e.g., psychologist, nursing assistant), describe their expertise, background and any specific training given. | The intervention content was developed by a health psychologist (HB) with expertise in health and work, a welfare officer, and a workplace health researcher. Development involved a co-creation process engaging 472 stakeholders. It is being delivered by a multidisciplinary research team with expertise in trial methodology, chronic pain, disability, and work.  The PAW Toolkit is accessed independently by participants. Support calls (or other accessible communications) with participants in the intervention group are provided by a Senior Occupational Therapist with chronic pain, and trials expertise. |
| **HOW** |  |
| Describe the modes of delivery (e.g., face-to-face or by some other mechanism, such as internet or telephone) of the intervention and whether it was provided individually or in a group. | The mode of delivery is remote via internet. The PAW Toolkit is accessed individually via a web link, from any PC or mobile device. |
| **WHERE** |  |
| Describe the type(s) of location(s) where the intervention occurred, including any necessary infrastructure or relevant features. | The PAW toolkit can be accessed at any time and in any location, according to user preferences. |
| **WHEN and HOW MUCH** |  |
| Describe the number of times the intervention is intended to be delivered and over what period of time, including the number of sessions, their schedule, and their duration, intensity or dose. | Users can choose the ‘actual schedule’ (the order of sections visited or re-visited), the ‘dose’ they receive (how much of the content they access), the ‘duration’ (how long they access it for), and the ‘intensity’ (how often they access it) of the intervention. Repeat visits are possible and encouraged. |
| **TAILORING** |  |
| If the intervention was planned to be personalised, titrated or adapted, then describe what, why, when, and how. | The PAW Toolkit is highly flexible to user needs and preferences, meaning that, although the content provided is the same for all users, the *way in which it is used* is highly personalised (as above).  Up to 3 telephone support calls - or other accessible model of communication - with the OT are taken at participant preference during the intervention period. This includes individually tailored advice and behavioural strategies for managing pain at work that is aligned to PAW content. The discussions are individually personalised to address specific needs of individual participants, with the specific nature of the discussion and advice provided recorded as part of the study. |
| **MODIFICATIONS** |  |
| If the intervention was modified during the course of the study, describe the changes (what, why, when, and how). | There are no plans to modify the intervention during the study. However, the results of this feasibility study will indicate whether modifications (to content or delivery approach) are required in a future trial or implementation study. |
| **HOW WELL** |  |
| Planned: If intervention adherence or fidelity was assessed, describe how and by whom, and if any strategies were used to maintain or improve fidelity, describe them. | Usage, fidelity, and engagement with the PAW Toolkit will be determined through collection of self-report data via a participant feedback form at T1, automated Google analytics at T1 and T2 and researcher-led interviews at T2. Fidelity of the OT support will be determined through researcher-led analysis of audio-recordings of a sub-sample of telephone calls (or other accessible communications) and forms completed by the OT at the time of each communication which will be synthesised at T1. SMS Text message reminders will be used to maximise engagement. |
