# Supplementary material for: The Web-Based Pain-at-Work Toolkit With Telephone Support for Employees With Chronic or Persistent Pain: Protocol for a Cluster Randomized Feasibility Trial
Source: JMIR Res Protoc. 2023 Oct 30;12:e51474. doi: 10.2196/51474 (PMC10644198; doi:10.2196/51474)
Supplement: Multimedia Appendix 3 [file resprot_v12i1e51474_app3.docx]

**Multimedia Appendix 3.** Mapping the PAW Toolkit intervention and feasibility trial to persuasive systems design

| **Category** | **Design technique** | **Description of use** |
| --- | --- | --- |
| Primary task support | Reduction | Toolkit information is presented in clear, simple language. |
|  | Tunnelling | Users are guided through a series of toolkit sections. |
|  | Tailoring | Users can view items that are relevant to their needs.  Telephone support is tailored to individuals’ needs. |
|  | Personalisation | Participants receive web link via personalised email.  Telephone support is personalised for participants. |
|  | Self-monitoring | Data collection involves self-monitoring of pain.  Users encouraged to take action to achieve goals (e.g., disability disclosure, discussion with managers, workplace adjustments, changes to lifestyle behaviours and pain coping strategies). |
|  | Simulation | Users can reflect on the direct influence of actions (e.g., lifestyle behaviours, work modifications) on pain and work ability. |
|  | Rehearsal | Videos to share stories from others with chronic pain. |
| Dialogue support | Praise | Participants thanked for intervention (and trial) engagement.  Positive feedback provided through telephone support. |
|  | Rewards | Opportunity to participate in a prize draw for completers. |
|  | Reminders | Text message reminders to access intervention and complete surveys. |
|  | Suggestion | Toolkit includes signposting to additional resources. Telephone support provides personalised suggestions. |
|  | Liking | Toolkit is visually appealing and considers issues of accessibility. |
|  | Social role | Toolkit presents advice and signposting for social actors (i.e., vocationally active adults with chronic pain). |
| Credibility support | Trustworthiness | Information and resources are truthful, fair and unbiased. This is verified through peer review. |
|  | Expertise | Toolkit content demonstrates expertise in subject area.  This is verified through stakeholder consultation and peer review during the development phase. |
|  | Surface credibility | Professional presentation, inclusion of logos for host organisation. |
|  | Real-world feel | Name of host organisation appears on front page, information about host organisation is publicly available. |
|  | Authority | Developers and all experts involved in development processes are named within the toolkit. |
|  | Third party endorsement | Collaboration with a pain charity – logo is included on front page. |
|  | Reliability | Periodical peer review to maintain currency of content. |
| Social support | Social learning | Video examples provide opportunity for observation of outcomes for others.  Vicarious reinforcement is anticipated within organisations participating in the trial (e.g., through raising awareness of chronic pain among employers and employees). |
|  | Social comparison | Video examples provide opportunity for social comparison.  Toolkit content provides opportunity to evaluate own attitudes, beliefs and behaviours towards chronic pain and its management at work. |
|  | Normative influence | Engagement with intervention will encourage personal agency, and reflection on normative influence of co-workers and managers on attitudes, beliefs and behaviours related to chronic pain and its management at work. |
|  | Social facilitation | Telephone support motivates participants to set personal goals and act on advice. |
|  | Cooperation | PAW Toolkit highlights the role of others in supporting people with chronic pain at work (e.g., ‘working together’, accessing support from line managers, government schemes etc) |
|  | Competition | Engagement in intervention does not involve competition. |
|  | Recognition | Developers and experts are recognised within the Toolkit.  Intervention and trial will raise awareness of chronic pain within workplace settings.  Participants receive individual recognition for engagement with intervention and completion of trial. |
