# Supplementary material for: The Web-Based Pain-at-Work Toolkit With Telephone Support for Employees With Chronic or Persistent Pain: Protocol for a Cluster Randomized Feasibility Trial
Source: JMIR Res Protoc. 2023 Oct 30;12:e51474. doi: 10.2196/51474 (PMC10644198; doi:10.2196/51474)
Supplement: Multimedia Appendix 4 [file resprot_v12i1e51474_app4.docx]

**Multimedia Appendix 4.** Feasibility and acceptability measures.

| Data collection, measurement methods, and details | | | | Purpose | T0^a^ | T1^b^ | T2^c^ |
| --- | --- | --- | --- | --- | --- | --- | --- |
| **Recruitment** | | | | | | | |
|  | **Study records** | | | | | |  |
|  |  | | The number of organizations and participants approached, consented, and randomized, the characteristics of those recruited any barriers to recruitment, and the timescale to recruit. | To assess whether it would be possible to recruit to a definitive trial. | ✓ |  |  |
| **Retention** | | | | | | | |
|  | **Study records** | | | | | |  |
|  |  | | The number of participants who complete outcome measures at T0, T1, and T2. | To assess whether it would be possible to retain participants in a definitive trial. | ✓ | ✓ | ✓ |
| **Intervention fidelity** | | | | | | | |
|  | **Participant feedback form** | | | | | |  |
|  |  | | Usage and perceptions of the intervention determined at T1. | To assess perceptions of the intervention and reported impacts on pain or work behavior change. |  | ✓ |  |
|  | **Google analytics** | | | | | | |
|  |  | | To collect data on fidelity (link distribution and receipt), frequency and intensity of engagement (initial access, page, and section views), duration of engagement (time spent using the Toolkit), type of engagement (active or passive). | To assess degree of engagement with the intervention and how this resource may be used in a definitive trial. |  | ✓ | ✓ |
|  | **Interviews (participants)** | | | | | | |
|  |  | | Usage and perceptions of the intervention determined at T2. | To explore perceptions of the intervention, any barriers/challenges and reported impacts on pain or work behavior change. |  |  | ✓ |
|  | **OT records and audio-recordings of a subsample of telephone calls (or other accessible communications).** | | | | | | |
|  |  | | Whether the OT^d^ support was conducted correctly and aligns with the PAW^e^ Toolkit. Whether applicable behavioral strategies and recommendations were delivered in relation to any problems identified and recorded. Record forms completed by the therapist at the time of each communication which will be synthesized at T1. | To assess whether OT support can be delivered consistently in a definitive trial. |  | ✓ |  |
| **Safety** | | | | | | | |
|  | **Study records** | | | | | |  |
|  |  | | Adverse event monitoring during the intervention period. | To assess the feasibility of safety data capture for a definitive trial. | ✓ | ✓ | ✓ |
| **Health care resource use** | | | | | | | |
|  | **Health economic questionnaire** | | | | | |  |
|  |  | | Items on TAU^f^ costs, health care resource use costs, medicines use, primary care consultations, hospital stays and outpatient visits for reasons related to chronic pain or anxiety or depression, and quality of life measurement. | To assess the feasibility of capturing health economic data in a future trial. | ✓ | ✓ | ✓ |
| **Acceptability** | | | | | | | |
|  | **Interviews (participants and stakeholders)** | | | | | |  |
|  |  | | Qualitative interviews will explore whether participants and employers find the intervention and trial design acceptable. | To assess the acceptability of using this intervention and design in a definitive trial. |  |  | ✓ |

^a^T0: time point (baseline).

^b^T1: time point 1 (3 months).

^c^T2: time point 2 (6 months).

^d^OT: occupational therapist.

^e^PAW: Pain-at-Work.

^f^TAU: treatment as usual.
