# Supplementary material for: The Web-Based Pain-at-Work Toolkit With Telephone Support for Employees With Chronic or Persistent Pain: Protocol for a Cluster Randomized Feasibility Trial
Source: JMIR Res Protoc. 2023 Oct 30;12:e51474. doi: 10.2196/51474 (PMC10644198; doi:10.2196/51474)
Supplement: Multimedia Appendix 5 [file resprot_v12i1e51474_app5.docx]

**Multimedia Appendix 5.** Employer- and participant-reported outcomes.

| Data collection, measurement methods, and details | | | | | T0^a^ | T1^b^ | T2^c^ |
| --- | --- | --- | --- | --- | --- | --- | --- |
| **Employer-reported outcomes** | | | | | | | |
|  | | | Data collected by web-based survey, email, and secure data transfer | |  |  |  |
| **Organization (at recruitment)** | | | | | | | |
|  | | **Web-based survey and email** | | | | |  |
|  | | |  | Employment setting characteristics: sector, type, and size, number of staff, role of the “gatekeeper” employee representative, organizations’ views toward organizations workplace culture, description of TAU^d^, existing provisions to support staff with long-term health conditions | ✓ | ✓ | ✓ |
| **Sickness absence rates** | | | | | | | |
|  | | **Employer records** | | | | |  |
|  | | | About participating employees (with consent) | | ✓ | ✓ | ✓ |
| **Participant-reported outcome measures** | | | | | | | |
|  | | | Data collected by web-based survey | |  |  |  |
| **Sociodemographic** | | | | | | | |
|  | | **Age (years)** | | | | |  |
|  | | |  | Continuous measure (≥18 y) | ✓ | ✓ | ✓ |
|  | | **Gender** | | | | |  |
|  | | |  | Female/male/prefer not to say/self-definition (FT^e^) | ✓ | ✓ | ✓ |
|  | | **Ethnic group** | | | | |  |
|  | | |  | White/Black- Caribbean/Black-African/Black-other/Indian/Pakistani/Bangladeshi/Chinese/Other (FT) | ✓ | ✓ | ✓ |
|  | | **Marital status** | | | | |  |
|  | | |  | Married or civil partnership/single/living with a partner/widowed/divorced/prefer not to say | ✓ | ✓ | ✓ |
|  | | **Living arrangements status** | | | | |  |
|  | | |  | Alone/with family or significant others/other | ✓ | ✓ | ✓ |
|  | | **School leaving age** | | | | |  |
|  | | |  | Continuous variable | ✓ | ✓ | ✓ |
|  | | **Direct transfer from school to full-time education/university** | | | | |  |
|  | | |  | Yes/no | ✓ | ✓ | ✓ |
|  | | **Educational attainment** | | | | |  |
|  | | |  | Five classes: GCSEs^f^, A-levels, vocational, university degree, professional | ✓ | ✓ | ✓ |
|  | | **Household income weekly total (in GBP^g^) (GBP £1, US $ 1.22)** | | | | |  |
|  | | |  | Eight classes: 0-99/ 100-149/ 150-249/ 250-349/ 350-449/ 450-599/ 600-749/≥750 | ✓ | ✓ | ✓ |
| **Health** | | | | | | | |
|  | **Pain (present)** | | | | | |  |
|  | | **Numerical rating scale [105]** | | | | |  |
|  | | |  | Continuous scale 0-10. “On a scale from 0 to 10, where 0 is no pain and 10 is the worst pain you’ve experienced, at this moment, what number represents your overall pain level?” | ✓ | ✓ | ✓ |
|  | **Pain duration** | | | | | |  |
|  | | | Years and months | | ✓ | ✓ | ✓ |
|  | **Comorbidities** | | | | | |  |
|  | | | Nine morbidities: present/absent/not sure. Morbidities: high blood pressure; heart problems; diabetes; kidney disease; stroke or TIA^h^; arthritis; asthma or lung conditions; anxiety or depression; liver or stomach problems; other (FT) | | ✓ | ✓ | ✓ |
|  | **Height and weight** | | | | | |  |
|  | | |  | Metric or imperial for calculation of BMI^i^ (weight [kg]/height^2^ [m]) | ✓ | ✓ | ✓ |
| **Employment characteristics from employee** | | | | | | | |
|  | | | Sector, size, and type of employing organization, assessment of their perception of organization culture, and which TAU services or support they have accessed via their employer (TAU may consist of [but is not limited to] any combination of the following: occupational health, counseling, line manager support, signposting to education about factors that may have positive or negative effects on chronic pain) | |  |  |  |
| **Current employment status.** | | | | | | | |
|  | | **WTI^j^ (part 1) [106]** | | | | |  |
|  | | |  | Twelve classes: full time/part-time/leave of absence/short-term sickness/long-term sickness/unemployed/not employed/retired/early retirement on grounds of ill health/homemaker/other (FT). Sick leave: start date. | ✓ | ✓ | ✓ |
|  | | **Job skill level 1-4 (UK standard Occupational Classification [107]** | | | | |  |
|  | | |  | Job title and industry and number of paid hours 1/52. Main and up to 3 other jobs | ✓ | ✓ | ✓ |
|  | | **Total hours and work pattern** | | | | |  |
|  | | |  | Average 1/52. Contracted and actual. Number of working days 1/7 | ✓ | ✓ | ✓ |
|  | | **Sole income status** | | | | |  |
|  | | |  | Yes/no | ✓ | ✓ | ✓ |
|  | | **Job sector classification [108]** | | | | |  |
|  | | |  | Standard Occupational Classification: 9 major classes | ✓ | ✓ | ✓ |
|  | | **Self-employment status** | | | | |  |
|  | | |  | Yes/no | ✓ | ✓ | ✓ |
|  | | **Employment duration** | | | | |  |
|  | | |  | Months and years | ✓ | ✓ | ✓ |
|  | | **Employee contract status** | | | | |  |
|  | | |  | Permanent/fixed term/0 h/agency/casual, freelance | ✓ | ✓ | ✓ |
|  | | **Self-employed role** | | | | |  |
|  | | |  | Sole trader/owner/freelancer/other (FT) | ✓ | ✓ | ✓ |
|  | | **Job responsibilities** | | | | |  |
|  | | |  | 9 items: 1-5 scale: work patterns and location | ✓ | ✓ | ✓ |
|  | | **Organization size** | | | | |  |
|  | | |  | Five classes: 1 person/micro (2-9)/ small (10-49)/ medium (50-249)/ large (≥250) | ✓ | ✓ | ✓ |
|  | | **Trade union membership (or similar)** | | | | |  |
|  | | |  | Yes/no/prefer not to say | ✓ | ✓ | ✓ |
|  | | **Occupational Health presence at main workplace** | | | | |  |
|  | | |  | Yes/no/do not know/not applicable | ✓ | ✓ | ✓ |
|  | | **Use of work-related support** | | | | |  |
|  | | |  | Eight classes: support and other (FT) in the last year | ✓ | ✓ | ✓ |
|  | | **Job satisfaction [109]** | | | | |  |
|  | | |  | 1 item: 1-5 scale to measure perceived job satisfaction | ✓ | ✓ | ✓ |
|  | | **Job stressfulness [111]** | | | | |  |
|  | | |  | 1 item: 1-5 scale to measure perceived job stress | ✓ | ✓ | ✓ |
|  | | **Sickness absence** | | | | |  |
|  | | |  | 1 item: 5 classes: number of sickness absence days in last year: no time/<5 d/5-20 d/>20 d/not working | ✓ | ✓ | ✓ |
|  | | **Turnover intentions [109]** | | | | |  |
|  | | |  | One item: yes/no | ✓ | ✓ | ✓ |
|  | | **Work ability index [111]** | | | | |  |
|  | | |  | One item: 0-10 rating: compared with lifetime best, current work ability | ✓ | ✓ | ✓ |
| **Work-related PROMs** | | | | | | | |
|  | **Presenteeism** | | | | | | |
|  | | **WLQ^k^ [112]** | | | | |  |
|  | | |  | 25 items: percentage time (in last 2/52) limited in: physical work demands, time demands, mental-interpersonal demands and output demands (0-4 scale of 0%-100%) | ✓ | ✓ | ✓ |
|  | **Work productivity and activity impairment** | | | | | | |
|  | | **WPAI:GH^l^ (V2.0) [113]** | | | | |  |
|  | | |  | Current employment status | ✓ | ✓ | ✓ |
|  | | |  | In last 1/52: number of hours missed due to health problems | ✓ | ✓ | ✓ |
|  | | |  | In last 1/52: number of hours missed not due to health problems | ✓ | ✓ | ✓ |
|  | | |  | In last 1/52: number of hours worked | ✓ | ✓ | ✓ |
|  | | |  | One item: rank 0-10: how much health problems affected work productivity, in last 1/52 | ✓ | ✓ | ✓ |
|  | | |  | One item: rank 0-10: how much health problems affected ability to perform regular daily activities, in last 1/52 | ✓ | ✓ | ✓ |
| **Work self-efficacy** | | | | | | | |
|  | | **WSE-S^m^ [114]** | | | | |  |
|  | | |  | Three items: rank 0-10: at present, confidence in: ability to work, manage condition at work, working not making condition worse | ✓ | ✓ | ✓ |
|  | | |  | Three items: rank 0-10: over next year, motivation to work, importance of continuing work, confidence of continuing work | ✓ | ✓ | ✓ |
| **Social support at work** | | | | | | | |
|  | | **DCSQ^n^ (Social Support at Work subscale) [115]** | | | | |  |
|  | | |  | 6 items: 1-4 scale. Measuring perceived atmosphere at work, relationship with and perceived support from coworkers and superiors | ✓ | ✓ | ✓ |
| **Psychological and health-related quality of life PROMs^o^** | | | | | | | |
|  | **Depression** | | | | | | |
|  | | **Patient health questionnaire [116]** | | | | |  |
|  | | |  | 2 items: 0-3 scale. Self-administered anxiety screening tool | ✓ | ✓ | ✓ |
|  | **Anxiety** | | | | | | |
|  | | **Generalized anxiety disorder [117]** | | | | |  |
|  | | |  | 7 items: 0-3 scale. Self-administered depression screening | ✓ | ✓ | ✓ |
| **Health-related quality of life** | | | | | | | |
|  | | **EQ-5D-5L [118]** | | | | |  |
|  | | |  | 5 items: 0-5 scale (mobility; self-care; usual activities; pain/discomfort; anxiety/depression; and health rating 0-100 scale) | ✓ | ✓ | ✓ |
| **Health resource use** | | | | | | | |
|  | **Use of NHS^p^ and Private Health Services** | | | | | | |
|  | | **Care from the GP^q^ surgery** | | | | |  |
|  | | |  | Number of visits in last 4/52: GP/practice Nurse/OT^r^/physiotherapist, other (FT) | ✓ | ✓ | ✓ |
|  | | **Complementary care** | | | | |  |
|  | | |  | Number of visits in last 4/52: chiropractor/osteopath/other (FT) | ✓ | ✓ | ✓ |
|  | | **Emergency NHS hospital admission for pain** | | | | |  |
|  | | |  | Number of days in last 4/52 | ✓ | ✓ | ✓ |
|  | | **Nonemergency NHS hospital admission for pain** | | | | |  |
|  | | |  | Planned hospital overnight stays in the last 6/12: department, number of nights | ✓ | ✓ | ✓ |
|  | | **Use of NHS hospital outpatient clinic appointments for pain** | | | | |  |
|  | | |  | Number of appointments in last 4/52. Department: rheumatology/orthopedics/pain management clinic/other (FT) | ✓ | ✓ | ✓ |
|  | | **Use of other NHS hospital clinic appointments for pain** | | | | |  |
|  | | |  | Number of appointments in last 4/52: OT/physiotherapist/other (FT) | ✓ | ✓ | ✓ |
|  | | **Use of private (non-NHS) hospitals for pain** | | | | |  |
|  | | |  | Number of days in last 4/52 | ✓ | ✓ | ✓ |
|  | | **Use of private (non-NHS) health care professionals for pain** | | | | |  |
|  | | |  | Number of visits in the last 4/52: doctor/OT/physiotherapist/chiropractor/osteopath/other (FT) | ✓ | ✓ | ✓ |
|  | | **Pain medication** | | | | |  |
|  | | |  | Name and number of days in last 4/52: prescribed/complementary/over the counter | ✓ | ✓ | ✓ |
| **Technology adoption PROMs (intervention only)** | | | | | | | |
|  | **PAW^s^ Toolkit intervention** | | | | | | |
|  | | **Technology acceptance questionnaire (TAM^t^) [99]** | | | | |  |
|  | | |  | 8 items: 1-5 scale: whether the PAW Toolkit increased knowledge and awareness, was relevant, easy to use, clear and understandable, changed attitudes, motivation, or help seeking behaviors to pain management at work |  | ✓ |  |
|  | | |  | Identify any barriers to using the PAW Toolkit (FT) |  | ✓ |  |
|  | | |  | Whether using the PAW Toolkit led to behavior change (FT) |  | ✓ |  |
|  | | |  | Whether the PAW Toolkit was useful and whether they would recommend to others |  | ✓ |  |
| **SMS text message reminders** | | | | | | | |
|  | | | Whether text reminders were acceptable, and whether they prompted access to the PAW Toolkit or the OT support | |  | ✓ |  |

^a^T0: time point (baseline).

^b^T1: time point 1 (3 months).

^c^T2: time point 2 (6 months).

^d^TAU: treatment as usual.

^e^FT: free-text option.

^f^GCSE: General Certificate of Secondary Education.

^g^GBP: Great British Pound.

^h^TIA: transient ischemic attack.

^i^BMI: Body Mass Index.

^j^WTI: Work Transitions Index.

^k^WLQ: Work Limitations Questionnaire.

^l^WPAI:GH: Work Productivity and Activity Impairment Questionnaire: general health.

^m^WSE-S: Work Self-Efficacy Scale.

^n^DCSQ: Demand Control Support Questionnaire.

^o^PROMs: Participant-Reported Outcome Measures.

^p^NHS: National Health Service.

^q^GP: general practitioner.

^r^OT: occupational therapist.

^s^PAW: Pain-at-Work.

^t^TAM: Technology Acceptance Model.
