# Supplementary material for: The Web-Based Pain-at-Work Toolkit With Telephone Support for Employees With Chronic or Persistent Pain: Protocol for a Cluster Randomized Feasibility Trial
Source: JMIR Res Protoc. 2023 Oct 30;12:e51474. doi: 10.2196/51474 (PMC10644198; doi:10.2196/51474)
Supplement: Multimedia Appendix 6 [file resprot_v12i1e51474_app6.pdf]

Reviewer 1

Individual Criteria	Comments
**Involvement of individuals with lived experience**	Given that the PaW tool was developed with MSK people involved, I would expect this to continue.
**Relevance**	It has the potential to be both relevant and useful. Whether it is depends on the take-up by employees and the support of employers.
**Approach**	Given that it builds on previous work, yes.
**Team**	The qualifications and positions held by the team members cannot be challenged. Their experience of these matters indicates that the budget is sound.
**Impact**	Given that it builds on previous work, yes.

Reviewer 2

Individual Criteria	Comments
**Relevance**	Strongly relevant to current policy interests, given the scale of the population of interest and the impacts to employees and employers
**Approach**	The question is researchable and the approach suitable in many ways. However, I feel further consideration (or explanation) is required as to:<br><br>• The role that employers/line managers will play – it is not clear to me. Will they just publicise the offer to staff, or be expected to engage with those utilising the support. And what about those in the control group? Particularly given the statement that some employees do not disclose their condition. I think there are potentially some ethical considerations here too.<br>• The necessity of the “control” group – why will those employers/employees engage with the study? How would this work in the proposed full-scale trial? Given the scale of the study, I am uncertain of the need for a control group at all (and they won’t be a “true” control group as will be engaged in the area of interest). I would envisage perhaps more important to have a fuller range of treatment sites. If it is to prove that it is possible to recruit one for a full-scale trial, I do not understand why there are the 30 minute conversations for employees.
**Team**	There is significant, strong expertise in the subject area. I am unclear on precisely who will take forward the different research elements – e.g. who has the relevant quant and qual work. However, have confidence that the senior team will appoint a suitable researcher. Given the three quantitative surveys and scale of qualitative work, I think it does offer value for money.
**Impact**	I think the dissemination does need some further consideration to have greatest impact. I think the investigators need to have greater clarity on what they wish to disseminate – is it the potential strength of the intervention? Or is it the fact that there is feasibility in running a trial. Or both?

Reviewer 3

Individual Criteria	Comments
**Relevance**	The global burden of chronic pain is growing with implications for both an ageing workforce and employers. The difficulties include both working people, trying to stay at work with chronic pain, and those wanting to return to work following sickness absence. Importantly, it is difficult to measure the impact of chronic pain on presenteeism/ work productivity, whilst people are at work. In 2016 it was estimated that ill health costs the UK economy £100 billion with musculoskeletal conditions being the second highest cause of sickness absence.<br><br>This is a highly relevant and timely study, proposing to test the feasibility of conducting a definitive cluster randomised controlled trial (RCT) of the effectiveness and cost-effectiveness of 'Pain at Work (PAW) Toolkit for working-age adults with chronic or persistent pain'. This web-based toolkit was co-created with 472 people who have pain, employers, and healthcare professionals (Blake et al, 2022) and pilot tested. It provides evidence-based education and advice about pain, disability rights, work capacity and adjustments, self-management strategies, and signposting to support (e.g., Access to Work scheme).<br><br>This study will determine whether it is feasible and acceptable to progress to a full-scale trial in the future. If shown to be effective, PAW could help people to better self-manage their pain. This may increase work participation and enjoyment, work productivity and overall quality of life, but also can help to contribute to reducing social inequalities and the overall health, societal and economic burden of chronic or persistent pain.
**Approach**	Both chronic pain management and work participation/ support are complex phenomenon to research and design interventions for. The applicants clearly appreciate this complexity and have had a robust methodological approach to co-create this tool with wide stakeholder involvement e.g., PPIE and clinicians and conducted a pilot testing. In the proposed feasibility and acceptability testing of this toolkit, they are proposing to recruit, over 12 months, working-age adults who have any type of chronic/persistent pain (including those with, or at risk of musculoskeletal conditions) and randomly allocate them to receive either the intervention (PAW Toolkit plus 3x30 minute telephone support calls from an occupational therapist including behavioural strategies for work-related self-management) or an active control comparator (usual advice from employer, plus 3x30- min non-specialist telephone check-in calls) via 8 workplaces, for up to 120 employees.<br><br>Study design is suitable and rigorous to include data collection of important work outcomes via questionnaires at 3 time points, (baseline, 3 and 6 months) and a qualitative study in which they plan to conduct ~40 interviews to explore their views about the PAW Toolkit and the trial, including employees from workplaces in the intervention group, and line managers or other people that employees identify as involved in their support at work.

**Team**	Very experienced and skilled study team, with specific/ relevant background in behavioural medicine, occupational medicine, rheumatology, health psychology, vocational rehabilitation, qualitative research, health economics and medical statistician. The research group also includes an advisory group with a widespread background and expertise and planning to meet through quarterly meetings. It is good to see a representation from National Health Improvement Lead for Public Health England and Chair for PHE's Muslim Network Collaboration (advocate for diversity and inclusion), a trade union rep, an expert in welfare and disability rights.<br><br>It is also noted that the applicants stated that a patient-partner will attend study meetings to input at every stage of the study across all 3 work-packages and facilitate access to further PPI input through pain charities, as Founder of Burning Nights Support Charity. They will recruit further lay members from local groups, including the Patient and Public Advisory Group (MSK PPAG) of the Pain Centre Versus Arthritis at the University of Nottingham and Aberdeen Epidemiology PPI group. However, there is no specific breakdown of the payments to patient research partners in the costing sheet, despite the confirmation that reimbursement of expenses will follow INVOLVE guidance. Unless this is covered under comms and dissemination (~£20K) or the study members section?
**Impact**	An impressive output and dissemination plan is included in the application, and it is positive to see that lay members will be given the opportunity to co-author manuscripts, present to PPI, contribute to writing study materials and dissemination materials.<br><br>If feasibility and acceptability are established, and progression criteria met, applicants aim to conduct a full-scale multicentre cluster randomised controlled trial to determine the intervention's effectiveness and cost-effectiveness. But most importantly, the wide stakeholder engagement in this study should help to raise awareness among employees about the prevalence of persistent pain in the workforce, and how they can manage their condition with support from their employer. Currently similar advice is only available from scattered resources in the NHS and the internet, as well as some arthritis charities, but they are not as readily accessible, relevant and/or specific to chronic pain management at work. Thus, this study has potential to have a positive impact on working people with chronic pain, as well as informing policy and practice.

Additional Comments:
Thank you for submitting your application titled 'The PAW Trial: feasibility and acceptability of the Pain-at- Work Toolkit' to the Oliver Bird Fund. I have read this study with much interest and was impressed with the wide stake holder involvement in the study. My question is about the PPIE in this study. Although there are extensive activities planned to involve and engage patient and public partners, I was unable to ascertain whether payment to patient partners is accounted for in the proposed budget. This may have been covered under a generic heading, but it is not explicitly stated. Clarity as to this would be much appreciated.
